# Self-reliance, Social Norms, and Self-stigma as Barriers to Psychosocial Help-Seeking Among Rural Cancer Survivors With Cancer-Related Distress: Qualitative Interview Study

**DOI:** 10.2196/33262

**Published:** 2022-05-19

**Authors:** Pamela Baker DeGuzman, David L Vogel, Veronica Bernacchi, Margaret A Scudder, Mark J Jameson

**Affiliations:** 1 School of Nursing University of Virginia Charlottesville, VA United States; 2 Department of Pyschology Iowa State University Ames, IA United States; 3 Department of Otolaryngology-Head and Neck Surgery School of Medicine University of Virginia Charlottesville, VA United States

**Keywords:** cancer survivorship, cancer-related distress, rural health, self-stigma, help-seeking, psychosocial referral, support networks, self-reliance

## Abstract

**Background:**

Even when technology allows rural cancer survivors to connect with supportive care providers from a distance, uptake of psychosocial referrals is low. Fewer than one-third of participants in a telemedicine intervention for identifying rural survivors with high distress and connecting them with care accepted psychosocial referral.

**Objective:**

The purpose of this research was to examine the reasons for which rural cancer survivors did not accept a psychosocial referral.

**Methods:**

We utilized a qualitative design to address the research purpose. We interviewed participants who had been offered psychosocial referral. Semistructured interviews were conducted 6 weeks later (n=14), and structured interviews were conducted 9 months later (n=6). Data were analyzed descriptively using an inductive approach.

**Results:**

Ultimately, none of the rural cancer survivors (0/14, 0%) engaged with a psychosocial care provider, including those who had originally accepted referrals (0/4, 0%) for further psychosocial care. When explaining their decisions, survivors minimized their distress, emphasizing their self-reliance and the need to handle distress on their own. They expressed a preference for dealing with distress via informal support networks, which was often limited to close family members. No survivors endorsed public stigma as a barrier to accepting psychosocial help, but several suggested that self-stigma associated with not being able to handle their own distress was a reason for not seeking care.

**Conclusions:**

Rural cancer survivors’ willingness to accept a psychosocial referral may be mediated by the rural cultural norm of self-reliance and by self-stigma. Interventions to address referral uptake may benefit from further illumination of these relationships as well as a strength-based approach that emphasizes positive aspects of the rural community and individual self-affirmation.

## Introduction

### Background

Cancer survivors from rural areas experience high-levels of cancer-related distress (the multifactorial, unpleasant, emotional experience that interferes with their ability to cope with cancer, treatment, and symptoms effectively). As recently as 10 years ago, fewer than 10% of all individuals with cancer were being screened for cancer-related distress, but with wider adoption of screening tools such as the National Comprehensive Cancer Network Distress Thermometer and Problem List [1], as many as 70% of individuals with cancer are now being screened [2-4]. Unfortunately, despite this high rate of screening, difficulty connecting rural survivors with psychosocial support persists. In fact, rates of successful referral of rural survivors to psychosocial services remains low [5-7], which leaves rural survivors highly vulnerable to a range of negative sequelae of unmet needs including a higher risk of suicide [8-11].

### Rural Head and Neck Cancer Survivorship

Survivors of head and neck cancer have unique posttreatment sources of distress that may profoundly impact quality of life. Cancers of the head and neck are the seventh most common worldwide, and the ninth in the United States; more than 53,000 US men and women were diagnosed with head and neck cancer in 2020 [12], and the number of diagnoses and deaths of head and neck cancer continue to increase, outpacing those of most other cancers in the United States. Over the past several decades, survival after head and neck cancer treatment has increased [13,14]; thus, a growing number of survivors live permanently impacted by treatment. Head and neck cancer affects areas of the body that are imperative for critical activities such as speech and swallowing. Patients who undergo surgery to remove cancer and surrounding tissue are left with lasting impacts on prominent and often noticeable areas of the tongue, throat, voice box, windpipe, or jawbone and cope with lasting pain, neck and shoulder dysfunction, dysphagia, speech changes, and deformities related to loss of facial integrity [15]. Research has shown that when cancer-related distress in survivors of head and neck cancer is not addressed, it persists far beyond the immediate posttreatment period—as many as 5 years after treatment, unaddressed needs persist, including pain, difficulty chewing and swallowing, depression, and anxiety [16,17]; these unaddressed impacts are highly and negatively correlated with survivors’ quality of life [17-19].

Unfortunately, rural survivors have more unmet emotional needs, significantly poorer health, and higher levels of psychological distress than their urban counterparts [20]. Ultimately, the sequalae of unmet needs likely contribute to the high suicide rate among head and neck cancer survivors, who are estimated to have the second and third highest rate of all persons with cancer [21], 3 to 4 times higher than that of the general population [22,23]. Given increasingly higher and widening risk of suicide for US rural residents, there is an urgent need to address the cancer-related distress of rural head and neck cancer survivors [24].

### Rural Head and Neck Cancer Survivors’ Low Uptake of Psychosocial Help

It is well accepted that distance from care is a significant factor in rural survivors’ reluctance to receive referral to psychosocial care [7,25,26]. Yet, this factor does not explain survivors’ unwillingness to receive psychosocial care when distance barriers can successfully be overcome via technology. For example, in a previous study [27,28], we developed and tested a telemedicine-delivered intervention for rural head and neck cancer survivors (called Comprehensive Assistance: Rural, Nursing Interventions and Guidance) to screen for cancer-related distress and make referrals for lingering posttreatment unmet needs. The intervention was designed to overcome technology barriers experienced by individuals living in broadband-poor areas, by offering options for individuals without home-based internet access to connect to a nurse with oncology specialization. Participants (n=14) who were found to have high cancer-related distress (using a combination of Distress Thermometer and Problem List measurement and a nurses assessment) were referred to a social worker with oncology specialization from the Cancer Center for psychosocial support, who initially contacted patients by phone to discuss the range of support options available (including telephone counseling or support groups in their area); however, fewer than one-third (28.6%) accepted referrals for further psychosocial care [27,28].

Given this low acceptance, even when distance barriers were removed, there is a need to better understand the psychological reasons why referrals are rejected and to develop interventions to increase acceptance; however, only one study [25] has directly examined rural cancer survivors’ reasons for not seeking psychosocial services—rural cancer survivors reported that speaking with a psychologist or using a support group to deal with psychosocial issues is not an accepted social norm. This suggested that relying on those within their own personal circle is more acceptable than utilizing professional care to deal with nonphysical issues that arise from cancer treatment [25]. Another study [29], in which men reported a desire to minimize or normalize the problem and to have emotional control noted the high value (in help-seeking in rural settings) that is placed on self-reliance and privacy.

It has also been suggested that stigma may be a barrier to seeking psychosocial services, especially within rural communities [30,31]. Stigma occurs when a person is labeled as less desirable than others for having an undesirable characteristic or trait (ie, a diagnosis cancer or mental illness) or engaging in an undesirable behavior (ie, seeking help). Stigma can occur at the external (public stigma) or at the internal (self-stigma) level [32]. Research specific to individuals with head and neck cancer has found low levels of public and self-stigma related to their cancer diagnosis [33], but the impact of stigma related to seeking psychosocial services has not been explored in rural head and neck cancer survivors. Higher rates of perceived public stigma for seeking help (ie, the perception that others view those who seek help for mental health concerns as weak or crazy) and self-stigma for seeking help (ie, the perception of oneself as being inferior or a failure for seeking help with a mental health concern) have been shown to exist in rural populations [31,32,34,35]. Self-stigma associated with seeking mental health concerns has been shown to affect the likelihood of newly diagnosed patients with head and neck cancer using psycho-oncology services [36]. However, past research has been largely limited to individuals not currently experiencing distress or who were given a hypothetical situation and asked about what they might do rather than measuring actual behavior or acceptance of service use. Research has also largely not focused on individuals currently experiencing distress when they are offered services. Given that only 28.6% of rural survivors with high cancer-related distress accepted referral for psychosocial care in our previous study [27], the purpose of this research was to directly examine the reasons rural cancer survivors accept or do not accept psychosocial referral.

## Methods

### Participants

We utilized a qualitative descriptive design [37] to accomplish the study purpose. Participants were patients who had been offered psychosocial referral during a telemedicine intervention [27]; these patients had been recruited from the head and neck cancer clinic at an National Cancer Institute–designated comprehensive cancer center in the southeastern United States that serves a large rural catchment area and were over 18 years of age, had completed active treatment for head and neck cancer within the past 3 months, and lived in a rural county (which we defined as small metropolitan, micropolitan, or noncore and at least 45 minutes of travel was required to reach the cancer center).

### Data Collection

Demographic, cancer-related, and level of distress data were collected as part of the previous study [27].

During open-ended semistructured interviews conducted (by a graduate nursing student under the supervision of the principal investigator; audiorecorded and transcribed verbatim) 6 weeks after the telemedicine intervention, participants who had declined a referral during the intervention were asked to talk about their reasons for declining, and those who had accepted the referral during the intervention were asked if they had yet heard from the referring provider and if the process had moved forward.

To understand perspectives on the barriers to acceptance of a psychosocial referral related to stigma or rural social norms, we attempted to recontact each participant who had been offered a psychosocial referral for a structured interview (9 months after the conclusion of the intervention). We used a structured interview guide and encouraged participants to expand on their answers in order to gain insight; interview questions were drawn from validated instruments [32,37] designed for understanding individuals’ reasons for not accepting or following through on referrals. Participants were asked if their decision to accept or not accept a referral was related to self-reliance (ie, feelings of not being able to take care of one’s own problems) [29], public stigma related to seeking mental health (ie, others viewing them negatively or in a less favorable light, others thinking bad things about them, or others seeing them as seriously disturbed or thinking they posed a risk to others), and self-stigma related to seeking mental health (ie, if accepting a psychosocial referral would impact them feeling “inadequate,” “inferior,” or “less satisfied with themselves” [32,38] (Multimedia Appendix 1). We also sought to determine if they had met with the Cancer Center social worker or other psychosocial care providers.

### Data Analysis

We used inductive content analysis to guide coding of the data. This methodology is appropriate for establishing links between the research objectives and the summary findings, ensuring that these links are transparent [39] and has been used in studies to identify characteristics of social media videos about college students’ mental health [40] and to guide the design of digital interventions for mental health management among construction personnel in Nigeria [37]. Two researchers collaboratively categorized data into known barriers to mental health care in rural populations. Categories were developed into themes; categories and themes were discussed among 3 members of the research team until consensus was reached.

### Ethics

The institutional review board for health research at the University of Virginia approved the study (HSR-IRB 20991). Verbal consent was obtained from all participants.

## Results

### Participant Characteristics

**Figure 1:**
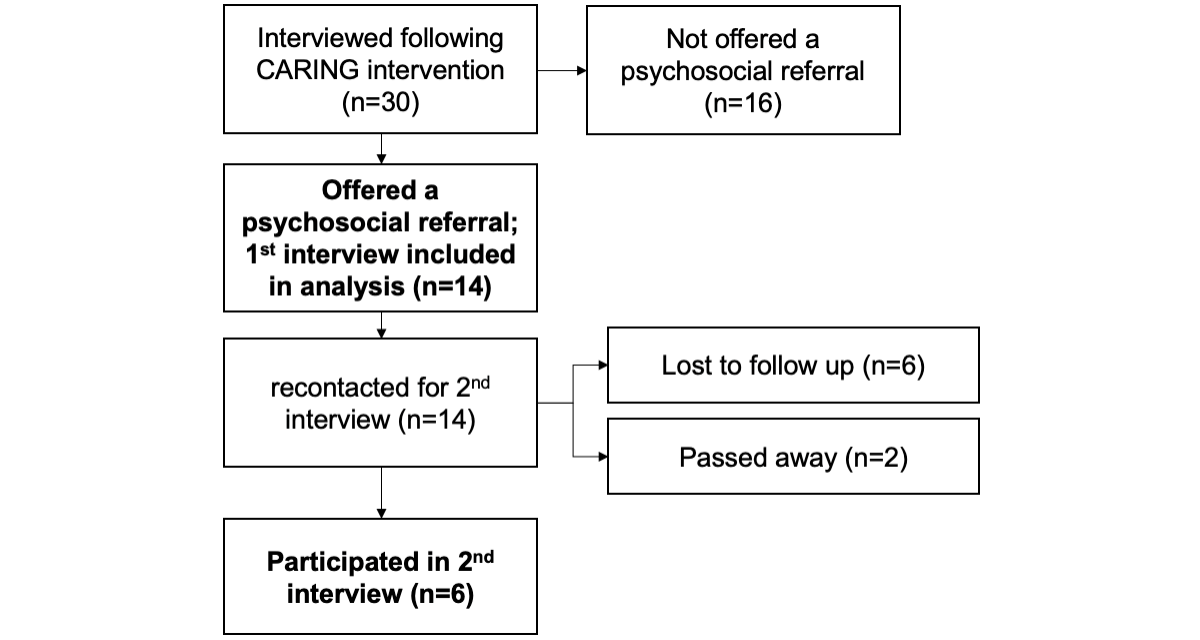
Participation flowchart. CARING: Comprehensive Assistance: Rural Interventions, Nursing, and Guidance.

All 14 individuals who had been referred for further psychosocial help had participated (Table 1) in the first round of interviews, and we were able to successfully recontact 6 of the original 14 for long-term follow-up interviews. Of the 14 participants, 2 had passed away since the intervention, and we were unable to reach 6 (Figure 1).

**Table 1:** Participant characteristics.

Characteristic			Participants (n=14)
**Gender, n (%)**			
	Male	7 (50)	
	Female	7 (50)	
Age (years), mean (SD)			62.0 (12.4)
**Race, n (%)**			
	White	11 (79)	
	Black	2 (14)	
	Asian or refused	1 (7)	
**Ethnicity, n (%)**			
	Non-Hispanic	12 (86)	
	Hispanic	2 (14)	
**Cancer site, n (%)**			
	Oral cavity	5 (36)	
	Thyroid	4 (29)	
	Pharynx	2 (14)	
	Other	2 (14)	
	More than one site	1 (7)	
**Cancer type, n (%)**			
	Squamous cell carcinoma	8 (57)	
	Papillary thyroid carcinoma	4 (29)	
	Other	2 (14)	
**Cancer stage, n (%)**			
	Early	12 (86)	
	Late	2 (14)	
Distress score^a^, mean (SD)			5.8 (0.3)

^a^The distress score was calculated as total number of problem areas rated as 4 or higher (out of 10); scores ranged from 5.17 to 7.17.

The sample was equally split among men and women; participants ranged in age from 39 to 80 years. The majority were White (11/14, 79%), non-Hispanic (12/14, 86%), and the most common cancer was squamous cell carcinoma of the oral cavity (5/14, 36%). Over 85% of participants (12/14) had cancer that had not spread to lymph nodes or metastasized elsewhere. The mean distress score was 5.8 (SD 0.3) out of 10. Of note, of the 6 survivors with whom we able to conduct second interviews, 2 women had originally accepted referrals, but when the social worker contacted them, neither had followed through with receiving further assistance.

### Themes

We ultimately extracted 3 themes: minimization and self-reliance; preference for use of informal support; and self-stigma for some but no public stigma.

#### Minimization and Self-reliance

When explaining their rationale for not pursuing further psychosocial care, 6 participants made light of their distress by minimizing it and instead focused on positivity and self-reliance. A positive attitude was viewed as a means to regain health:

Yeah well that’s the only way to get better, isn’t it?...I certainly try not to ever feel sad. I feel lucky [that the cancer is in remission].

70-year-old male

Several laughed while describing their distress. One participant who had declined to speak with a social worker insisted that his situation was not negative and that he could and should handle it himself:

I had a brain bleed. They drilled a couple of holes in my head. But life goes on! There was uh...a series of complications with the cancer. This is just a series of misfortunes the way I look at it [laughs], and I have to deal with them.

80-year-old male

He then emphasized his self-reliance and strength by stating,

And I do deal with them...I’m not an invalid or anything.

One woman minimized her fear of recurrence, continuously reframing her fears of recurrence as concerns, stating

it’s just those same concerns. You know I get this sore throat and it’s like, it’s just a concern that the cancer may come back like the other time. So, it’s just a concern that it’ll come back like the other two times...it’s not even the fear it’s just the concern of it, of it coming back.

33-year-old female

The idea of cancer-related distress not rising to the level of requiring outside intervention was echoed by other survivors. One woman who declined a referral to the Cancer Center social worker stated,

I just tend to view things I want to handle them myself...If I felt an absolute need [to speak to someone about my cancer-related distress] I would do it...I didn’t feel an urgent need, to be honest.

71-year-old female

#### Preference for Use of Informal Support

Participants described a small circle of people with whom they would speak about their cancer-related distress. They reported limiting these discussions to close family but occasionally included close friends. One turned down the referral to the Cancer Center social worker by explaining

my niece is a social worker and we’ve had our chats.

52-year-old female

Two men reported only discussing distress with their spouses. When asked why he had not been interested in speaking with the Cancer Center social worker, one replied,

No. I’ve got great support from my wife as a caregiver.

80-year-old male

and the other reported that he spoke to his wife about his cancer-related distress, but only minimally.

I'm uh, I’m just not a big talker [laughs]. My wife’s always gettin’ on me. That’s just uh, just kinda the way I am.

62-year-old male

One woman expressed a similar sentiment:

What you might consider an inadequacy, or a problem or an issue, I handle them myself, and that’s just the way I am...It would take some getting beyond the point of me handling it entirely on my own and feeling comfortable in sharing with someone else.

71-year-old female

However, she also suggested that her family was included as part of her handling things herself:

My family is close, husband is very close...those are my anchors.

#### Self-stigma for Some but No Public Stigma

Participants did not report public stigma associated with seeking help for cancer-related distress. One woman who reported high distress during the intervention but had turned down a referral to the social worker stated,

I don't think anyone would say anything bad about [my speaking to a social worker].

52-year-old female

Despite participants’ sense that society would not judge them harshly, a few of them did indicate they would judge themselves negatively if they had followed up with a social worker or other mental health care provider. One woman who had originally accepted a referral, but then never called the social worker back, was asked if speaking to the social worker would have made her feel that she could not handle her problems herself, and she confirmed,

I would question it...I don’t want to learn [sic] the appearance that as I’m getting older, I am less able to handle my situation, and that’s a protection on my part. I try not to view anybody else that way, but I tend to view myself more critical [sic].

71-year-old female

Not all survivors shared this perception. One participant with high distress who turned down the social worker referral stated that he did not believe would not have felt inadequate if he had accepted help from the social worker:

I wouldn’t think there was any stigma to it.

80-year-old male

Another participant explained that she had not accepted a referral to the social worker because

I have a therapist.

50-year-old female

She went on to explain her perspective of the impact of socioeconomic status on her views about seeking mental health care:

I make over $100,000, you know, I have a bunch of degrees and everything...but I do live in a rural area...I think for me there aren’t those barriers to treatment.

Despite many survivors’ statements suggesting that seeking mental health help for cancer-related distress is acceptable, overwhelmingly participants still declined the opportunity to speak to a social worker or counselor. During the intervention, one man had self-reported high levels of nervousness and worry related to his cancer diagnosis, treatment, and the possibility of recurrence, but he declined the offer of psychosocial support from a social worker or participation in a support group. During the follow-up interview, he reported feeling no public or self-stigma associated with seeking help for his cancer-related distress but was unable to articulate his reasons for refusing, stating

The social worker thing was something that I did not feel would help me.

62-year-old male

## Discussion

### Principal Findings

When explaining their views toward not accepting psychosocial help, head and neck cancer survivors minimized their experiences of distress while emphasizing self-reliance and a desire to only speak to close family and occasionally to friends. Research has found similar phenomena—cancer survivors living in rural populations preferred to rely on family and friends to deal with psychosocial issues [25]. Several studies [29,31,41-43] have also found self-reliance and problem-minimization to be barriers to seeking mental health treatment for rural populations. Similarly, in a qualitative study [30], psychosocial care providers serving a rural Australian region reported that the distress of residents of their region had to rise to a very serious threshold before residents would even acknowledge the existence of mental health distress. Both their findings [30] and our findings suggests that the rural social norms of self-reliance and desire to not share personal information with someone from outside the survivors’ immediate circle may be key contributing factors to not seeking help for psychological distress among rural cancer survivors, as well as important points to address when developing an intervention. This is particularly important, as prior research indicates that mental health care discussions with only family and friends are insufficient to address the profound distress that survivors experience and that those who elect to seek professional care find it highly impactful [44].

Consistent with past research [29], both male and female survivors in our study emphasized positive thinking, self-reliance, and minimization of distress; our findings also suggested that there were some differences between men and women in approaches to addressing barriers to care. Men in our study reported a smaller circle of trust, which typically only included their wives, which is consistent with the findings of a study with 409 rural Australian men and women which found that rural men reported more barriers to seeking mental health care than women [29]. Certain norms, such as a desire for stoicism and emotional control, have been found to be stronger barriers to help-seeking for rural men than they are for women [42]. Because women who live in rural areas are more likely to seek mental health care than men who live in rural areas [42,45], approaches to overcome barriers may benefit from gender-focused interventions, for example, a dyadic intervention that includes their spouse or caregiver may be particularly salient for men.

Interestingly, while stigma is the most cited barrier to seeking help, rural cancer survivors in our study showed that the type of stigma was especially important. Self-stigma was a barrier to accepting a referral; public stigma was not strongly felt. None of the 6 individuals reported that others would view them negatively if they were to seek help from the Cancer Center social worker. In turn, self-stigma associated with seeking psychosocial services was found to be a factor that limited the acceptance of a psychosocial referral, at least for some participants. These findings are consistent with assertions that stigma is a moderately important barrier to help-seeking, which have been reported by one-quarter to one-third of participants in multiple research studies included in a review [46] and in a study of patients newly diagnosed with head and neck cancer [36]. One possible explanation is that the cancer experience is viewed to be sufficiently physically and emotionally debilitating to warrant emotional support from others but still does not rise to a level that warrants breaking social norms oneself. One participant referred specifically to this, stating that she tried not to view others that way but viewed herself more critically. This finding is also consistent with most research showing that self-stigma is a more salient barrier to help-seeking than public stigma in general populations [47] and rural communities [30,32]. As such, self-stigma may be an important barrier for those whose values are aligned with not seeking psychosocial help, and thus, is not salient for everyone.

Alternatively, it may be that stigma influences other factors, and thus, its systemic effects may not always be noticeable [48]. Others have also suggested that stigma is part of interrelated network of barriers [49]. For example, Jennings and colleagues [50] examined a model and linked public stigma to attitudes toward seeking professional services through the mediators *self-stigma* and *self-reliance*, which, being proximal to decisions to seek help, may be more accessible to participants’ awareness. This finding is consistent with our theme *preference for informal support* that potential mediating factors such as self-reliance and the desire to only disclose to close family and friends were widely endorsed and stigma factors less so. Thus, similar to Jennings et al [50], we encourage researchers and clinicians to continue to examine the complex relationships between different types of stigma and other factors such as self-reliance in order to be able to develop more focused interventions to increase the use of services by those who could benefit.

### Implications for Cancer Survivorship Care

Our findings suggest a potential direction for developing interventions aimed at improving access to psychosocial care for rural cancer survivors. Barriers to rural access are typically grouped into 4 domains: people, place, provider, and payment [51]. In light of increased levels of insurance coverage [51], and ongoing technological advances that overcome distance-related barriers to care [52], personal and cultural belief systems need to be explored further. Thus, to continue to work toward equitable access, a more rigorous understanding of rural cultural belief systems that may limit cancer survivors’ openness to receiving high-quality psychosocial support is needed. Instruments are available that differentiate barriers [32,38,53]; however, these tools were not developed specifically with rural populations; thus, customization to illuminate rural-specific barriers may be needed. As such, researchers may consider amending instruments by including questions specific to rural populations.

Clinicians caring for rural survivors may need to be aware if they hold any negative perception of or implicit bias toward rural culture. Current approaches toward improving access to care have been developed in the context of an urban health care delivery system [54]. For example, rather than viewing aspects of rural culture through a deficit lens (ie, stigma and self-reliance as barriers to access), we should strive to develop culturally appropriate interventions that leverage the considerable strengths of the rural setting (ie, resilience, strong community networks) to design effective interventions that connect rural survivors with care [55].

A strength-based approach that might be particularly salient is one based in self-affirmation theory [56-58]. Self-affirmation theory notes that we are inherently motivated to keep a positive sense of self-worth, and when we experience information that could decrease positive self-perceptions (ie, reduce the belief that we are self-reliant and self-sufficient), we are driven to protect positive views of the self, which can result in avoidance of treatment for mental health issues [59,60]. Fortunately, self-affirmation theory also asserts that we can reduce this drive to protect our self-worth, and thus increase likelihood of seeking therapy, by using self-affirmations (ie, reflecting on a positive and self-relevant personal characteristic or values) prior to confronting information that could decrease these positive self-perceptions. Self-affirmation interventions have started to receive some support for increasing the use of psychosocial services [59-61] but need to be further evaluated with rural cancer survivors. This approach, if tailored to this population, may be able to directly reduce barriers to access such as stigma and self-reliance.

### Study Limitations

Our study was qualitative, and as such, findings were meant to provide a direction for further research and were not meant to be generalizable. Still, there are several limitations that may have had an impact on our findings. The sample size (n=14) was small, and we were only able to recontact 6 individuals to discuss specific barriers. It is possible that with a larger sample size we may have found additional themes. The number of participants also made it difficult to fully evaluate differences in perceptions between rural women and men, or between people with different types of stages of cancer. Finally, we did not collect data on participants’ education level, which is a significant driver of differences in stigma perceptions [62] and was also highlighted by one participant. Evaluating our findings in the context of education level and cancer stage may have provided additional insight into drivers of participants' reluctance to seek psychosocial help. Researchers should explore the impact of stigma and self-reliance on psychosocial referral uptake using quantitative instruments and with a larger sample size, to better understand which populations may be experiencing these impacts. More precise information about how self-reliance and stigma create barriers to help-seeking will be important in developing customized interventions for rural individuals and should be considered when targeting behavioral change in rural populations.

### Conclusions

To the best of knowledge, this study is the first explore self-reliance, rural social norms, and self-stigma as barriers to connecting rural survivors with psychosocial care. Our findings suggest that rural cancer survivors who experience these barriers may be reluctant to seek psychosocial care, even when they identify themselves as having high levels of cancer-related distress. Further research, with a larger sample, to explore these barriers is needed to develop effective interventions to increase psychosocial referral uptake in this population.

## Appendix

Multimedia Appendix 1 Interview questions.

## References

[1] Holland JC, Andersen B, Breitbart WS, Buchmann LO, Compas B, Deshields TL, Dudley MM, Fleishman S, Fulcher CD, Greenberg DB, Greiner CB, Handzo GF, Hoofring L, Hoover C, Jacobsen PB, Kvale E, Levy MH, Loscalzo MJ, McAllister-Black R, Mechanic KY, Palesh O, Pazar JP, Riba MB, Roper K, Valentine AD, Wagner LI, Zevon MA, McMillian NR, Freedman-Cass DA (2013). Distress management. J Natl Compr Canc Netw.

[2] Carlson M, Booth K, Byrnes E, Paul C, Fradgley E (2020). Pin-pointing service characteristics associated with implementation of evidence-based distress screening and management in australian cancer services: data from a crosssectional study. J Psychosoc Oncol Res Pract.

[3] Skaczkowski G, Sanderson P, Shand M, Byrne A, Wilson C (2018). Factors associated with referral offer and acceptance following supportive care problem identification in a comprehensive cancer service. Eur J Cancer Care (Engl).

[4] Skaczkowski G, Pejoski N, Kaur J, White V, Livingston PM, Wilson C (2020). Distress and problem assessment among people living with cancer from culturally and linguistically diverse backgrounds. Psychooncology.

[5] Bauwens S, Baillon C, Distelmans W, Theuns P (2014). Systematic screening for distress in oncology practice using the distress barometer: the impact on referrals to psychosocial care. Psychooncology.

[6] Funk R, Cisneros C, Williams RC, Kendall J, Hamann HA (2016). What happens after distress screening? patterns of supportive care service utilization among oncology patients identified through a systematic screening protocol. Support Care Cancer.

[7] Ervin K, Opie C, Koschel A, Jeffreson L, Haines H (2019). Unmet supportive care needs of rural men with cancer: a qualitative study. Austral J Cancer Nurs.

[8] DeGuzman P, Colliton K, Nail C, Keim-Malpass J (2017). Survivorship care plans: rural, low-income breast cancer survivor perspectives. Clin J Oncol Nurs.

[9] Deleemans JM, Mothersill K, Bultz BD, Schulte F (2020). Ethical considerations in screening head and neck cancer patients for psychosocial distress. Support Care Cancer.

[10] Hubbard G, Venning C, Walker A, Scanlon K, Kyle RG (2015). Supportive care needs of women with breast cancer in rural Scotland. Support Care Cancer.

[11] Tzelepis F, Paul CL, Sanson-Fisher RW, Campbell HS, Bradstock K, Carey ML, Williamson A (2018). Unmet supportive care needs of haematological cancer survivors: rural versus urban residents. Ann Hematol.

[12] Siegel RL, Miller KD, Jemal A (2020). Cancer statistics, 2020. CA Cancer J Clin.

[13] Pulte Dianne, Brenner Hermann (2010). Changes in survival in head and neck cancers in the late 20th and early 21st century: a period analysis. Oncologist.

[14] Noone A, Howlader N, Krapcho M, Miller D, Brest A, Yu M, Ruhl J, Tatalovich Z, Mariotto A, Lewis DR, Chen HS, Feuer EJ, Cronin KA (2018). SEER cancer statistics review, 1975-2015. National Cancer Institute.

[15] Murphy BA, Ridner S, Wells N, Dietrich M (2007). Quality of life research in head and neck cancer: a review of the current state of the science. Crit Rev Oncol Hematol.

[16] Wells M, Cunningham M, Lang H, Swartzman S, Philp J, Taylor L, Thomson J (2015). Distress, concerns and unmet needs in survivors of head and neck cancer: a cross-sectional survey. Eur J Cancer Care (Engl).

[17] Cramer JD, Johnson JT, Nilsen ML (2018). Pain in head and neck cancer survivors: prevalence, predictors, and quality-of-life impact. Otolaryngol Head Neck Surg.

[18] So WKW, Choi KC, Chen JMT, Chan CWH, Chair SY, Fung OWM, Wan RWM, Mak SSS, Ling WM, Ng WT, Yu BWL (2014). Quality of life in head and neck cancer survivors at 1 year after treatment: the mediating role of unmet supportive care needs. Support Care Cancer.

[19] Henry M, Habib L, Morrison M, Yang JW, Li XJ, Lin S, Zeitouni A, Payne R, MacDonald C, Mlynarek A, Kost K, Black M, Hier M (2013). Head and neck cancer patients want us to support them psychologically in the posttreatment period: survey results. Pall Supp Care.

[20] Weaver KE, Geiger AM, Lu L, Case LD (2013). Rural-urban disparities in health status among US cancer survivors. Cancer.

[21] Zaorsky Nicholas G, Zhang Ying, Tuanquin Leonard, Bluethmann Shirley M, Park Henry S, Chinchilli Vernon M (2019). Suicide among cancer patients. Nat Commun.

[22] Zeller JL (2006). High suicide risk found for patients with head and neck cancer. JAMA.

[23] Misono S, Weiss NS, Fann JR, Redman M, Yueh B (2008). Incidence of suicide in persons with cancer. J Clin Oncol.

[24] Ivey-Stephenson AZ, Crosby AE, Jack SPD, Haileyesus T, Kresnow-Sedacca M (2017). Suicide trends among and within urbanization levels by sex, race/ethnicity, age group, and mechanism of death - United States, 2001-2015. MMWR Surveill Summ.

[25] Andrykowski MA, Burris JL (2010). Use of formal and informal mental health resources by cancer survivors: differences between rural and nonrural survivors and a preliminary test of the theory of planned behavior. Psychooncology.

[26] Pesut B, Robinson CA, Bottorff JL, Fyles G, Broughton S (2010). On the road again: Patient perspectives on commuting for palliative care. Pall Supp Care.

[27] DeGuzman PB, Vogel DL, Horton B, Bernacchi V, Cupp CA, Ghamandi BJF, Hinton ID, Sheffield C, Jameson MJ (2021). Examination of a distress screening intervention for rural cancer survivors reveals low uptake of psychosocial referrals. J Cancer Surviv.

[28] DeGuzman PB, Bernacchi V, Cupp CA, Dunn B, Ghamandi BJF, Hinton ID, Jameson MJ, Lewandowski DL, Sheffield C (2020). Beyond broadband: digital inclusion as a driver of inequities in access to rural cancer care. J Cancer Surviv.

[29] Fennell K, Hull M, Jones M, Dollman J (2018). A comparison of barriers to accessing services for mental and physical health conditions in a sample of rural Australian adults. Rural Remote Health.

[30] Fuller J, Edwards J, Procter N, Moss J (2000). How definition of mental health problems can influence help seeking in rural and remote communities. Aust J Rural Health.

[31] Schroeder S, Tan CM, Urlacher B, Heitkamp T (2021). The role of rural and urban geography and gender in community stigma around mental illness. Health Educ Behav.

[32] Vogel DL, Wade NG, Haake S (2006). Measuring the self-stigma associated with seeking psychological help. Journal of Counseling Psychology.

[33] Lebel S, Castonguay M, Mackness G, Irish J, Bezjak A, Devins GM (2013). The psychosocial impact of stigma in people with head and neck or lung cancer. Psychooncology.

[34] Stewart H, Jameson JP, Curtin L (2015). The relationship between stigma and self-reported willingness to use mental health services among rural and urban older adults. Psychol Serv.

[35] Sandelowski M (2000). Whatever happened to qualitative description?. Res Nurs Health.

[36] Cohen A, Ianovski LE, Frenkiel S, Hier M, Zeitouni A, Kost K, Mlynarek A, Richardson K, Black M, MacDonald C, Chartier G, Rosberger Z, Henry M (2018). Barriers to psychosocial oncology service utilization in patients newly diagnosed with head and neck cancer. Psychooncology.

[37] Vogel DL, Wade NG, Ascheman PL (2009). Measuring perceptions of stigmatization by others for seeking psychological help: Reliability and validity of a new stigma scale with college students. J Couns Psychol.

[38] Thomas DR (2016). A general inductive approach for analyzing qualitative evaluation data. Am J Eval.

[39] Choi B, Kim H, Huh-Yoo J (2021). Seeking mental health support among college students in video-based social media: content and statistical analysis of YouTube videos. JMIR Form Res.

[40] Nwaogu J, Chan A, Naslund J, Hon C, Belonwu C, Yang J (2021). Exploring the barriers to and motivators for using digital mental health interventions among construction personnel in Nigeria: qualitative study. JMIR Form Res.

[41] Judd F, Jackson H, Komiti A, Murray G, Fraser C, Grieve A, Gomez R (2006). Help-seeking by rural residents for mental health problems: the importance of agrarian values. Aust N Z J Psychiatry.

[42] Komiti A, Judd F, Jackson H (2006). The influence of stigma and attitudes on seeking help from a GP for mental health problems: a rural context. Soc Psychiatry Psychiatr Epidemiol.

[43] Snell-Rood C, Hauenstein E, Leukefeld C, Feltner F, Marcum A, Schoenberg N (2017). Mental health treatment seeking patterns and preferences of Appalachian women with depression. Am J Orthopsychiatry.

[44] Gunn K, Turnbull D, McWha JL, Davies M, Olver I (2013). Psychosocial service use: a qualitative exploration from the perspective of rural Australian cancer patients. Support Care Cancer.

[45] Wrigley S, Jackson H, Judd F, Komiti A (2005). Role of stigma and attitudes toward help-seeking from a general practitioner for mental health problems in a rural town. Aust N Z J Psychiatry.

[46] Clement S, Schauman O, Graham T, Maggioni F, Evans-Lacko S, Bezborodovs N, Morgan C, Rüsch N, Brown JSL, Thornicroft G (2014). What is the impact of mental health-related stigma on help-seeking? a systematic review of quantitative and qualitative studies. Psychol Med.

[47] Vogel DL, Wade NG, Hackler AH (2007). Perceived public stigma and the willingness to seek counseling: the mediating roles of self-stigma and attitudes toward counseling. J Couns Psychol.

[48] Clement S, Brohan E, Jeffery D, Henderson C, Hatch SL, Thornicroft G (2012). Development and psychometric properties the Barriers to Access to Care Evaluation scale (BACE) related to people with mental ill health. BMC Psychiatry.

[49] Schomerus G, Angermeyer MC (2008). Stigma and its impact on help-seeking for mental disorders: what do we know?. Epidemiol Psichiatr Soc.

[50] Jennings KS, Cheung JH, Britt TW, Goguen KN, Jeffirs SM, Peasley AL, Lee AC (2015). How are perceived stigma, self-stigma, and self-reliance related to treatment-seeking? a three-path model. Psychiatr Rehabil J.

[51] Newkirk IV, Damico A (2014). The Affordable Care Act and insurance coverage in rural areas. The Henry J Kaiser Family Foundation.

[52] DeGuzman PB, Jain N, Loureiro CG (2021). Public libraries as partners in telemedicine delivery: a review and research agenda. Pub Libr Q.

[53] Brenner RE, Colvin KF, Hammer JH, Vogel DL (2021). Using item response theory to develop revised (SSOSH-7) and ultra-brief (SSOSH-3) self-stigma of seeking help scales. Assessment.

[54] Mohatt N, Mohatt D, Benuto Lorraine T, Duckworth Melanie P, Masuda Akihiko, O'Donohue William O (2020). Rural prejudice-urban bias: the stories and structures that oppress rural communities. Prejudice, Stigma, Privilege, and Oppression.

[55] Bernacchi V, Zoellner J, Keim-Malpass J, DeGuzman P (2021). Rural resilience in cancer survivors: conceptual analysis of a global phenomenon. Online J Rural Nurs Health Care.

[56] Sherman DK, Cohen GL (2006). The psychology of self-defense: self-affirmation theory. Adv Exp Soc Psychol.

[57] Sherman D, Hartson K, Alicke MD, Sedikides C (2011). Reconciling self-protection with self-improvement. Handbook of Self-Enhancement and Self-Protection.

[58] Steele CM (1988). The psychology of self-affirmationustaining the integrity of the self. Adv Exp Soc Psychol.

[59] Lannin DG, Guyll M, Vogel DL, Madon S (2013). Reducing the stigma associated with seeking psychotherapy through self-affirmation. J Couns Psychol.

[60] Seidman AJ, Lannin DG, Heath PJ, Vogel DL (2019). Setting the stage: The effect of affirming personal values before psychotherapy intake screenings on perceptions of self-stigma and self-disclosure. Stigma Health.

[61] Seidman AJ, Wade NG, Lannin DG, Heath PJ, Brenner RE, Vogel DL (2018). Self-affirming values to increase student veterans' intentions to seek counseling. J Couns Psychol.

[62] Jones Arden R, Cook Trevor M, Wang Jianli (2011). Rural-urban differences in stigma against depression and agreement with health professionals about treatment. J Affect Disord.

