# Supplementary material for: Self-reliance, Social Norms, and Self-stigma as Barriers to Psychosocial Help-Seeking Among Rural Cancer Survivors With Cancer-Related Distress: Qualitative Interview Study
Source: JMIR Form Res. 2022 May 19;6(5):e33262. doi: 10.2196/33262 (PMC9164097; doi:10.2196/33262)
Supplement: Multimedia Appendix 1 [file formative_v6i5e33262_app1.docx]

**Interview Questions**

| 1. On (date), you participated in our telemedicine study with a nurse from the Cancer Center. During that visit, the nurse offered to refer you to a social worker [substitute other provider throughout as necessary]. You (accepted/declined) that referral. 2. **If they accepted the original referral**: did you ever speak with the social worker? (For either yes or no) do you recall why you chose to (meet/not meet) with the provider? 3. **If they did not accept the original referral**: Do you recall why you chose not to accept a referral to a social worker?   **STIGMA EVALUATION**  “Next, I’m going to ask you to think about some factors in your decision to (accept/not accept) a referral to a social worker. Please respond to each of these items as *not at all, a little*, *some, a lot,* or *a great deal*. To what degree do you believe that the people you interact with would __ if you had accepted a referral.”   1. ﻿React negatively to you 2. Think bad things of you 3. See you as seriously disturbed 4. Think of you in a less favorable way 5. Think you posed a risk to others 6. Think you could not handle your problems yourself   “You responded that the people you interact with might ______ . Can you tell me more about your thoughts that people might react this way?”  **SELF STIGMA EVALUATION**  “Next, I’m going to ask you to think about some factors in your decision to (accept/not accept) a referral to a social worker. ﻿People at times find that they face problems that they consider seeking help for. This can bring up reactions about what seeking help would mean. Please use the following choices to rate the degree to which each item impacted your decision: *strongly disagree, disagree, agree/disagree equally, agree, strongly agree”*  *﻿*   1. ﻿I would feel inadequate if I spoke to a social worker for help. 2. ﻿It would make me feel inferior to ask a social worker for help. 3. ﻿If I spoke to a social worker, I would be less satisfied with myself. 4. I would feel like I could not handle my problems myself   “You indicated ______ may have affected your decision to (accept/not accept) a referral. Can you tell me more about your thoughts that people might react this way? |
| --- |
